# Glial Modulator Antibiotics for Neuropathic Pain: Current Insights and Future Directions

**DOI:** 10.3390/ph18030346

**Published:** 2025-02-28

**Authors:** Alex J. Zimmerman, Nicholas Mangano, Grace Park, Amit K. Kaushal, Sergio D. Bergese

**Affiliations:** 1 Department of Physical Medicine and Rehabilitation, Stony Brook Medicine, Stony Brook, NY 11794, USA; alex.zimmerman@stonybrookmedicine.edu; 2 Department of Anesthesiology, Stony Brook Medicine, Stony Brook, NY 11794, USA; nicholas.mangano@stonybrookmedicine.edu; 3 Renaissance School of Medicine, Stony Brook University, Stony Brook, NY 11794, USA; grace.park@stonybrookmedicine.edu; 4 Chronic Pain Division, Department of Anesthesiology, Stony Brook Medicine, Stony Brook, NY 11794, USA; amit.kaushal@stonybrookmedicine.edu

**Keywords:** glial, neuropathic pain, minocycline, doxycycline, ceftriaxone, azithromycin

## Abstract

Pathological pain is defined as pain that outlives its usefulness as a protective warning system and becomes debilitating, disrupting normal life function. Understanding the mechanism of transition from physiological to pathological pain is essential to provide the effective prevention of chronic pain. The main subcategories of pathological pain are nociceptive pain, neuropathic pain, and nociplastic pain. Glial cells play pivotal roles in the development and maintenance of each of these pathological pain states, specifically neuropathic pain. Consequently, targeting these cells has emerged as a promising therapeutic strategy, as limited efficacy and harmful adverse effects are associated with current pharmacotherapies. This paper aims to review specific antibiotics that modulate glial cells, which can be used to treat neuropathic pain. These antibiotics include minocycline, doxycycline, ceftriaxone, and azithromycin. The potential of these antibiotics appears promising, particularly given the extensive prior research and use of these antibiotics in humans for other illnesses. However, each presents its own set of limitations, ultimately making the translation from preclinical findings to human therapies for neuropathic pain challenging.

## 1. Introduction

Pain is an essential, primitive protective mechanism designed to serve as a warning system in the setting of actual or potential tissue damage. It originates from a physiological process triggered by a peripheral stimulus. Failure to resolve this stimulus can lead to peripheral and central sensitization, which in turn leads to the consequent development of pathological pain (Dinakar & Stillman, 2016 [1]; Fregoso et al., 2019 [2]; Cheng, 2018 [3]).

Neuropathic pain, a pathological pain condition, is a dysfunction of the somatosensory nervous system. Neuropathic pain has various etiologies, that include nerve compression or systemic diseases that affect the peripheral and central nervous system, such as diabetes or autoimmune diseases. Depending on the location of the inciting lesion, neuropathic pain can further be classified as peripheral or central neuropathic pain (Xu et al., 2019 [4]; Wei et al., 2019 [5]; Ara & Maqbool, 2022 [6]). While numerous studies have explored the factors that initiate and perpetuate neuropathic pain, recent research has focused on the role of glial cells in both the pathogenesis and resolution of this pain state.

Glial cells are non-neuronal cells found in both the central and peripheral nervous systems. Glial cells contribute to both the development and maintenance of pain states. Two key types of glial cells, microglia and astrocytes, have been shown to significantly influence pain pathways (Tan et al., 2021 [7]). Microglia are the resident immune cells of the central nervous system. In settings of trauma, infection, or neurodegenerative diseases, microglia contribute to neuroinflammation by releasing cytokines, chemokines, and other proinflammatory metabolites (Magni et al., 2024 [8]; Paolicelli et al., 2022 [9]). These inflammatory mediators include prostaglandin E2, tumor necrosis factor-α (TNF-α), interleukin-1β (IL-1β), and nerve growth factor. These mediators are known to stimulate and amplify signaling, leading to increased pain sensitivity (Julius & Basbaum, 2001 [10]; Scholz & Woolf, 2007 [11]; Baral et al., 2019 [12]; Breitinger & Breitinger, 2023 [13])**.** Astrocytes similarly play a critical role in maintaining the neuronal environment by producing cytokines and chemokines, regulating synaptic transmission, and promoting interactions between glial cells and neurons through their neuronal receptors (Wang & Xu, 2022 [14]). In the presence of IL-1β and other proinflammatory mediators, the inflammatory cascade triggers astrocytes to activate local neurons and other glial cells, and the ongoing crosstalk among these cells leads to sustained neuroinflammation and pathological pain states (Gajtkó et al., 2020 [15]).

Given their contributions to neuroinflammation, both microglia and astrocytes play critical roles in the onset and persistence of neuropathic pain (Jha et al., 2012 [16]) and, therefore, are promising targets for therapeutic intervention. While neuropathic pain has traditionally been treated with medications such as opioids, antidepressants, anticonvulsants, topical medications, and local anesthetics (Bannister et al., 2020 [17]), there is emerging evidence that suggests that antibiotics like minocycline, doxycycline, ceftriaxone, and azithromycin exert neuroprotective effects by modulating glial activity (Garrido-Mesa et al., 2013 [18]). The non-antibiotic actions of these drugs offer the opportunity to repurpose antibiotics as innovative therapies to target glial-mediated neuroinflammation and treat neuropathic pain. These mechanisms, as well as the fairly limited preclinical and clinical data, will be further explored.

## 2. Target Glial Modulators

### 2.1. Minocycline

Minocycline, a broad-spectrum antibiotic belonging to the tetracycline class, exerts its antibiotic properties primarily through the inhibition of bacterial protein synthesis by binding and inactivating the bacterial ribosome’s 30S subunit (Cunha et al., 1982 [19]). Minocycline has also exhibited the inhibition of the nuclear factor kappa-light-chain-enhancer (NF-κB) signaling pathway in activated B cells. NF-κB activation in glial cells is a common mechanism through which proinflammatory cytokines are produced (Sivamaruthi et al., 2023 [20]). Minocycline can modulate autophagy, a cellular process that helps clear damaged organelles and proteins during stress conditions. It has also been shown to inhibit the expression of inducible nitric oxide synthase (iNOS) and cyclooxygenase-2, enzymes involved in the production of neurotoxic molecules like nitric oxide and prostaglandins. This can mitigate mitochondrial dysfunction in glial cells by reducing oxidative stress (Lima et al., 2012 [21]). More recent in vitro studies have identified specific molecular targets of minocycline that may be related to its action in models of neuropathy. In the PC12 cell line, which is a common rat pheochromocytoma cell used to model neurodegenerative injury, minocycline was associated with the phosphorylation of the p47phox regulatory subunit and the subsequent anti-apoptotic inhibition of the MAPK pathway (Eslami et al., 2024 [22]).

Minocycline has been well studied in preclinical trials using mice and rat models, emerging as a therapeutic candidate for neuropathic pain (Zhou et al., 2018 [23]). In a study on adult female rats with chronic constriction injury (CCI) of a nerve, Vanelderen et al. studied the effects of intravenous amitriptyline, gabapentin, and minocycline on neuropathic pain behavior and spinal brain-derived neurotrophic factor (BDNF) expression, a protein key to the survival of brain and spinal cord nerve cells. Treatment with minocycline and amitriptyline significantly reduced thermal hyperalgesia, suggesting their potential clinical relevance for neuropathic pain treatment. However, gabapentin did not show similar effects. In addition, only minocycline reduced BDNF when compared to amitriptyline and gabapentin. These findings illustrate the potential differences in the modes of action of these drugs and the clinical need for minocycline to be tested for the treatment of neuropathic pain (Vanelderen et al., 2013 [24]).

Building upon these findings, Zarei et al. further investigated minocycline’s efficacy in reducing neuropathic pain after the ligation of the sciatic nerve in adult male rats. While intravenous minocycline showed promise in preventing neuropathic pain when administered before nerve injury, it failed to alleviate established neuropathic pain when given afterward. This highlights the importance of timing in minocycline administration and suggests that its effectiveness may vary depending on the stage of neuropathic pain development (Zarei et al., 2017 [25]). Interestingly, through analyses of adult male rat models of induced spinal root avulsion, performed by Chin et al., a dose-dependent quality of minocycline regarding its beneficial effect was discovered. When minocycline was given intraperitoneally, microglial proliferation was inhibited, but there was an increase in motor neuron survival. The inhibitory effect was enhanced by intrathecal minocycline, as the targeted drug delivery approach increased the bioavailability of the therapeutic agent. However, at higher concentrations, motor neuron survival was compromised with the intrathecal route due to the more efficient drug delivery. This demonstrated that the optimal form of administration involved a moderate intrathecal dose (Chin et al., 2017 [26]).

Transitioning from animal studies to human trials, research by Vanelderen et al. (trial number: NCT01869907) explored the efficacy of oral minocycline and amitriptyline in treating lumbosacral radicular pain in male and female humans between the ages of 18 and 80 yrs old. Overall, both drugs demonstrated potential benefits over two weeks, with minocycline showing fewer adverse events. However, questions about the clinical significance arose due to small effect sizes, emphasizing the need for larger, longer-term trials to assess their true impact on neuropathic pain in humans (Vanelderen et al., 2015 [27]).

In a randomized controlled trial by Martinez et al. (trial number 2008-001803-41) evaluating 100 patients undergoing lumbar discectomy, oral minocycline was administered for eight days prior to surgery. The primary endpoints were the incidence and severity of leg or back pain at 3 months postoperatively. The analysis revealed that the minocycline administration had no significant impact, as there was no difference in the incidence or severity of neuropathic pain and functional scores between the minocycline and placebo groups. The exploratory analysis suggested that minocycline might be effective in a subgroup of patients with predominantly deep spontaneous pain at baseline (Martinez et al., 2013 [28]). However, perioperative minocycline administration for eight days did not improve persistent pain after lumbar discectomy. The study, which was well powered and remains one of the few inquiries into the perioperative administration of minocycline for analgesic purposes, may reinforce the importance of establishing adequate glial modulation before the anticipated neurologic insult (Martinez et al., 2013 [28]).

Given the limited number of human trials exploring glial modulation by minocycline, a clinical trial (trial number NCT03106740) has been launched to evaluate its potential in relieving chronic low back pain (cLBP) in humans. The trial is titled “Assessing the potential anti-neuroinflammatory effect of minocycline in chronic low back pain: Protocol for a randomized, double-blind, placebo-controlled trial”. This ongoing trial aims to target neuroinflammation as a treatment avenue for cLBP using integrated Positron Emission Tomography/Magnetic Resonance Imaging (PET/MRI) scans to assess levels before and after treatment (Morrissey et al., 2023 [29]).

### 2.2. Doxycycline

Doxycycline is another antibiotic that has been implicated in the modulation of glial activity. Like minocycline, doxycycline is a second-generation tetracycline that acts as a bacteriostatic antibiotic by inhibiting protein translation by bacterial ribosomes, namely by binding the acceptor site on the 70S ribosome (Cunha et al., 1982 [19]). As early as 1992, doxycycline was found to exhibit non-antibiotic activity by affecting the proliferation of endothelial and glial cells (Guerin et al., 1992 [30]). That activity was later understood to be primarily related to the modulation of gene expression, and genes encoding glial-specific proteins were also found to be induced by doxycycline. Specifically, the in vitro expression of glial-derived neurotrophic factor (GDNF) and tyrosine hydroxylase (TH) was demonstrated to be tightly controlled by the addition of a doxycycline solution at different concentrations (Wang et al., 2006 [31]). Neurotrophic factors, such as GDNF, are versatile proteins that can be expressed in both physiologic and pathologic states and have been implicated in both maintenance and regenerative processes in neurons (Nakamura et al., 2011 [32]). In a 2013 study involving rat models of transected sciatic nerves, Schwann cells with doxycycline-inducible GDNF genes were injected into the site of a nerve injury. Upon doxycycline administration, the rats exhibited the elevated expression of GDNF in both the injured nerve and in the dorsal root ganglion cells, compared to rats administered with a placebo, and after 3 weeks, the nerves exhibited dose-dependent regeneration. Interestingly, at greater doses of administered doxycycline, the nerves regenerated into a disorganized tangle of cells, emphasizing the value of the strict translational regulation of GDNF (Shakhbazau et al., 2013 [33]). More recently, the direct binding of doxycycline to the pituitary adenylate cyclase-activating polypeptide (PACAP) receptor protein was demonstrated in vitro and resulted in the intracellular internalization of the doxycycline-PACAP receptor complex. This was concurrently associated with in vivo anti-apoptotic and anti-oxidative neuroprotective responses by cells treated with doxycycline in response to an external insult, a known effect of PACAP receptor activation (Yu et al., 2015 [34]).

The utility of doxycycline as a modulator of glial cell protein expression is not limited to that of GDNF. Pathogen-associated molecular patterns, such as lipopolysaccharide (LPS), have been shown to act on glial cells and induce the phosphorylation and activation of the mitogen-activated protein kinase (MAPK) and NF-κB molecular signaling pathways. These proinflammatory cascades result in the expression of an extracellular release of neuroinflammatory cytokines, TNF-a and IL-1b (Zhang et al., 2013 [35]). These cytokines promote a protective immune response and tissue repair and remodeling in the acute phase but can result in neurodegenerative changes in chronic states of release (Cunningham, 2013 [36]). In mouse models treated with LPS, the coadministration of doxycycline resulted in a significant decrease in glial activation, marked by the inhibition of the MAPK and NF-κB cascades and the decreased expression of TNF-a and IL-1b (Santa-Cecília et al., 2016 [37]). While this study involved mouse models of Parkinson’s disease and demonstrated decreased levels of striatal neurodegeneration as the primary outcome, the mechanism involving the doxycycline-induced downregulation of proinflammatory glial cytokines suggested broad applicability.

Doxycycline has also demonstrated a role in blunting the proinflammatory signaling cascades that follow exposure to oxidative stress. In mouse microglial cells, treatment with doxycycline resulted in a significant reduction in the intracellular reactive oxygen species (ROS), which was accompanied by decreased intracellular glucose and NADPH (Junior et al., 2021 [38]). Because NADPH oxidase, which produces ROS for oxidative burst responses in proinflammatory states, requires NADPH synthesis from glucose, Junior et al. suggest that glycolysis inhibition is another possible co-occurring mechanism by which doxycycline exerts its anti-inflammatory property in microglia. This hypothesis was strengthened by the observation that 2-deoxy-D-glucose, a potent inhibitor of glycolysis, yielded similar reductions in ROS production (but not in inflammatory cytokine release) by microglia (Fodelianaki et al., 2019 [39]).

More recent studies of non-antibiotic derivatives of tetracyclines, including the doxycycline derivative DDOX, have also shown efficacy in the reduction of nociceptive pain. DDOX, as well as the demeclocycline-derivative DDMC, showed significant analgesic properties when injected in mouse models of formalin-induced nociceptive pain (Nascimento et al., 2024 [40]). These findings, which are limited to mouse models but notably include the noninferiority of a single dose of DDMC compared to a single dose of morphine, point to tetracyclines and their derivatives as possible opioid alternatives.

### 2.3. Ceftriaxone

Ceftriaxone is a third-generation cephalosporin that was developed in 1978 for the treatment of Gram-positive and Gram-negative bacteria, acting as a bactericidal b-lactam antibiotic by cross-linking bacterial peptidoglycans to inhibit bacterial cell wall synthesis (Auer & Weibel, 2017 [41]).

In in vitro models of neuronal injury and degeneration due to glutamate excitotoxicity, ceftriaxone demonstrated the stimulated expression of the glial glutamate transporter GLT1. This, along with a concomitant increase in GLT1 activity, was associated with delays in neurodegeneration (Rothstein et al., 2005 [42]). Chronic neuropathic pain, which has been associated with GLT1 downregulation in the spinal cord, became a target of investigation into the potential antinociceptive properties of ceftriaxone. In an early study by Hu et al., mouse models of neuropathic pain following sciatic nerve injury were exposed to ceftriaxone or sham treatments. Intrathecal ceftriaxone was associated with the reversal of thermal hyperalgesia and mechanical allodynia, along with GLT1 upregulation and increased glutamate uptake in the dorsal horn of the spinal cord (Hu et al., 2010 [43]). Chen et al. demonstrated similar findings in opioid-induced hyperalgesia mouse models, wherein the downregulation of GLT1 due to opioid-induced hyperalgesia was reversed by ceftriaxone (Chen et al., 2012 [44]). Similar findings were noted by Gunduz et al. when mouse models of diabetic peripheral neuropathy were treated with ceftriaxone and exhibited a reduction in hyperalgesia and allodynia. This effect was reversed by the administration of the GLT1-inhibitor dihydrokainic acid, further associating glutamate neurotoxicity (and its amelioration by ceftriaxone) with neuropathic pain (Gunduz et al., 2011 [45]). The physiology of glutamate neurotransmission in neuropathic pain was further elucidated in 2012, when Inquimbert et al. measured miniature excitatory postsynaptic currents (mEPSCs) in the dorsal horn neurons of rats before and after peripheral nerve injury. These signals markedly increased in frequency after injury, and cerebrospinal glutamate concentrations also increased and remained elevated for up to 4 weeks. The inhibition of postsynaptic glutamate receptors reversed these changes, as did treatment with ceftriaxone (Inquimbert et al., 2012 [46]). The applicability of ceftriaxone to different models of neuropathic pain has been accentuated by recent studies, including one by Luo et al. that related ceftriaxone administration to the recovery of once-downregulated GLT1 receptors in mouse models of trigeminal neuropathy (Luo et al., 2020 [47]).

Ceftriaxone’s involvement in modulating oxidative stress and apoptosis signaling was also implicated in a 2014 study involving rat models of nerve injury. The expression of proapoptotic proteins Bax, Bcl2, caspase 3, and caspase 9, were measured before and after treatment with ceftriaxone. Oxidative stress markers, namely malondialdehyde and glutathione, were also measured before and after treatment. Compared to treatment with saline, treatment with ceftriaxone resulted in decreases in those proapoptotic proteins, as well as in oxidative stress markers (Amin et al., 2014 [48]).

The analgesic properties of ceftriaxone, which were well established in models of neuropathic pain, were also shown to be amplifiable by the coadministration of minocycline. In mouse models of nerve injury, reductions in thermal hyperalgesia were enhanced by adding minocycline to ceftriaxone, in a manner that exceeded the summation of their independent effects (Amin et al., 2015 [49]). Similarly, in mouse models subjected to the ligation of spinal nerves with the subsequent development of neuropathic pain, the addition of the PPARg-agonist pioglitazone to ceftriaxone resulted in the synergistic potentiation of their individual analgesic properties (Pottabathini et al., 2016 [50]). These combinatorial studies underscore the multifactorial pathophysiology of neuropathic pain; ceftriaxone is experimentally effective at treating one of the components.

As early as 2013, there have been case reports of cephalosporin treatment resulting in the resolution of pain in humans. In 2013, a case of postoperative nociceptive pain was effectively treated with ceftriaxone (Macaluso et al., 2013 [51]). In 2014, a woman with treatment-refractory complex regional pain syndrome (CPRS) received sustained symptom relief with treatment with cefadroxil, a cephalosporin derivative. This effect was noted to last for at least 3 years, throughout which, her symptoms returned with any discontinuation of cefadroxil (Ware & Bennett, 2014 [52]). However, translating the animal studies to specifically evaluate the efficacy of cephalosporins in treating neuropathic pain syndromes in humans (e.g., CPRS) remains an untapped opportunity.

### 2.4. Azithromycin

Discovered in the 1940s as antibiotic alternatives to b-lactams, macrolides (such as azithromycin) inhibit bacterial protein synthesis by binding to and deactivating the 50S subunit of bacterial ribosomes. Soon after their discovery, macrolides demonstrated non-antibiotic anti-inflammatory properties in disease states ranging from asthma to bronchiectasis, and research in the 1960s uncovered their inhibition of proapoptotic pathways and of proinflammatory cytokine and ROS production (Shinkai et al., 2008 [53]). Given the known anti-inflammatory properties of azithromycin, Amantea et al. applied azithromycin to mouse models of ischemic stroke to investigate neuroprotective effects. This 2016 study identified dose-dependent reductions in brain infarct size and neurological deficits after azithromycin administration. They also identified a shift in microglial polarization after the administration of azithromycin, from the proinflammatory M1 to the anti-inflammatory M2 phenotype (Amantea et al., 2016 [54]).

This neuroprotection was similarly demonstrated in mouse models of spinal cord injury (SCI), in which the administration of azithromycin after injury resulted in improvements in neuronal recovery and the associated increased ratio of the expression of M2 to M1 phenotypic markers on microglial cells harvested from the injured spinal cord (Gensel et al., 2017 [55]). Further research determined that functional improvement (e.g., locomotion) was also significantly increased in mouse models of SCI that received azithromycin immediately after injury. However, these models exhibited no difference in the rates of developing neuropathic pain (Kopper et al., 2019 [56]). Interestingly, in mouse models of chronic SCI with existing neuropathic pain in the form of heat-induced hyperalgesia, the administration of high-dose azithromycin significantly reduced this pain (Gensel et al., 2019 [57]). These data suggest that azithromycin can modulate existing neuropathic pain, rather than the pathophysiologic processes involved in its initial development secondary to SCI. Conversely, in a more recent study of SCI in rats, a 3-day course of azithromycin administered 30 min after injury demonstrated significant improvements in multiple behavioral measures of neuropathic pain (i.e., mechanical allodynia and heat hyperalgesia) while redemonstrating reductions in inflammatory cytokine expression and the M1:M2 microglial phenotype ratio (Rismanbaf et al., 2022 [58]).

Mice subjected to optic nerve injuries also exhibited improvements in retinal ganglion cell (RGC) survival following treatment with azithromycin. These mice also exhibited reductions in microglia activation, rates of RGC apoptosis, and the expression of several inflammatory biomarkers, including Bax, IL-1b, NF-κB, and superoxide dismutase 1 (Zloto et al., 2022 [59]). While not directly supporting the efficacy of azithromycin in neuropathic pain models, this finding (which was independently substantiated by Mahaling et al.) further supports the broad neuroprotective function of azithromycin in peripheral neurotrauma, one of the known mechanisms of neuropathic pain (Mahaling et al., 2022 [60]). However, the specific molecular target to which azithromycin binds and modulates these apoptotic molecular signaling pathways has yet to be elucidated.

## 3. Discussion

Minocycline, doxycycline, ceftriaxone, and azithromycin show promising potential for treating neuropathic pain. These antibiotics share the ability to influence glial cell activity and modulate neuroinflammatory pathways. However, despite their promise, each presents its own set of limitations, ultimately making their translation from preclinical findings to human therapies challenging. Below are key limitations and considerations:

### 3.1. Drug Specificity for Glial Cells

In addition to their direct effects on glial cells, antibiotics like minocycline also have various non-glial effects, which may limit their specificity for glial cell targets in neuropathic pain. Recent in vitro studies have identified specific molecular targets of minocycline that could be related to its action in neuropathy models, including the PC12 cell line (Eslami et al., 2024 [22]). For instance, in a study on rat dorsal root ganglion (DRG) neurons, minocycline was found to inactivate Na+ currents and accelerate the recovery of these currents from inactivation (Kim et al., 2010 [61]). Similarly, in another PC12 cell study, doxycycline promoted neurite outgrowth through the activation of the trkA receptor and downstream signaling pathways (PI3K/Akt and MAPK/ERK), while also enhancing the expression of key proteins such as GAP-43, synapsin I, and NF200, which are critical for axonal and synaptic plasticity (Amaral et al., 2021 [62]). In Alzheimer’s disease rat models, ceftriaxone was shown to simultaneously target the proteostasis network, neuroinflammation, glutamate excitotoxicity, oxidative pathways, and neurotrophic function (Tikhonova et al., 2021 [63]). Additionally, a study of COVID-19 patients revealed that ceftriaxone had multiple immunomodulatory effects, including the inhibition of proinflammatory cytokine production, the suppression of neutrophil influx, the induction of regulatory functions in macrophages, and alterations in autophagy (Venditto et al., 2021 [64]).

### 3.2. Timing and Stage of Pain Development

The timing of administration plays a critical role in the efficacy of these drugs, particularly in the early stages of injury and/or neuroinflammation. In animal studies, minocycline is more effective when administered before or shortly after nerve injury (Zarei et al., 2017 [25]) but fails to alleviate established neuropathic pain when administered long after injury. In rat models of nerve regeneration, doxycycline administered in the early stages of injury led to the elevated expression of GDNF and subsequent regeneration (Shakhbazau et al., 2013 [33]). In spinal cord in vitro models, ceftriaxone administered early in the neuroinflammatory process led to a larger upregulation of GLT1 and improvement in glutamate uptake reducing oxidative stress on nerves (Rothstein et al., 2005 [42]). In SCI in vitro models, azithromycin administered immediately post-injury led to significant improvements in locomotion and neuronal recovery (Gensel et al., 2017 [55]). This suggests that these antibiotics might be most effective during the early stages of injury and/or neuroinflammation. However, further research into their later administration needs to be studied, as their benefits might diminish once the neuropathic pain becomes chronic. This limitation emphasizes the importance of identifying the correct therapeutic window for intervention.

### 3.3. Dose Dependency and Side Effects

All four of the antibiotics demonstrate dose-dependent effects, with higher doses increasing the risk of adverse outcomes. For minocycline, higher doses have been shown to impair motor neuron survival (Chin et al., 2017 [26]). Similarly, higher doses of doxycycline can disrupt nerve regeneration (Shakhbazau et al., 2013 [33]). Ceftriaxone and azithromycin are generally safe at therapeutic doses, but prolonged use or high concentrations may cause toxicity. In addition, azithromycin could potentially cause side effects with long-term use at higher doses due to its broader immunomodulatory effects. These drugs require careful dosing to balance efficacy with safety. This highlights the complexity of dosing and the potential for adverse outcomes, particularly in delicate neurological contexts.

### 3.4. Challenges in Drug Delivery and Bioavailability

Drug delivery and bioavailability present challenges for all these antibiotics. Minocycline and doxycycline are more effective when delivered intrathecally, although this method comes with the risk of toxicity at higher concentrations. Ceftriaxone, being a cephalosporin, requires parenteral administration and faces challenges in crossing the blood–brain barrier. However, studies have shown that it can effectively target the spinal cord and produce neuroprotective effects (Rothstein et al., 2005 [42]). Azithromycin, which crosses the blood–brain barrier, has demonstrated neuroprotective effects in models of ischemic stroke, SCI, and optic nerve injury (Zloto et al., 2022 [59]), suggesting that it has the potential to treat neuroinflammation in the central nervous system (CNS). This highlights the challenge of optimizing drug delivery to achieve the desired therapeutic effect without causing harm.

### 3.5. Limited Human Data

Although minocycline and doxycycline have been tested in several human trials with some success in treating neuroinflammatory conditions, the lack of large-scale, multicenter studies makes it difficult to draw definitive conclusions on their efficacy in neuropathic pain. Ceftriaxone has been less studied in human clinical trials for neuropathic pain, although isolated case reports suggest it might be effective in chronic pain conditions such as postoperative pain and complex regional pain syndrome (Macaluso et al., 2013 [51]); Ware & Bennett, 2014 [52]). Azithromycin has even-more-limited human clinical data for treating neuropathic pain, but preclinical findings support its potential, particularly in conditions involving ischemic stroke, SCI, and optic nerve injury (Amantea et al., 2016 [54]; Zloto et al., 2022 [59]). Further clinical research is needed to fully assess the efficacy and safety of these compounds in human neuropathic pain.

### 3.6. Clinical Trial Design Issues

Most of the clinical studies have involved small sample sizes or short treatment durations, which makes it difficult to draw definitive conclusions regarding long-term efficacy and safety. For example, minocycline seemed to show some promise in specific subgroups of patients, such as those with predominantly deep spontaneous pain (Martinez et al., 2013 [28]). This suggests that its effectiveness may not be universal, and identifying which subgroups would benefit most from minocycline could be challenging. The variability in patient response and the difficulty in predicting who will benefit from treatment are significant hurdles in translating preclinical findings to clinical trials and then the broader patient population. Lastly, most clinical trials have been short-term, often limited to a few weeks. Chronic pain conditions are long-lasting, and the effects of these antibiotics over extended periods remain uncertain, especially given that it is an antibiotic with potential for bacterial resistance or other adverse outcomes when used chronically.

### 3.7. Competing with Established Treatments

While these antibiotics have shown some potential in reducing neuropathic pain, FDA-approved medications for neuropathic pain have more established efficacy profiles (please refer to Table 1, highlighting several guideline medications and their mechanism of action to treat neuropathic pain). This makes it harder for these antibiotics to compete unless they can demonstrate clear and consistent superiority in clinical settings, which has not yet been proven.

## 4. Conclusions

Minocycline, doxycycline, ceftriaxone, and azithromycin each offer unique mechanisms of action that modulate neuroinflammation and glial cell activity, making them potential treatments for neuropathic pain. However, challenges such as optimal dosing, timing, drug delivery, and a lack of extensive human data need to be addressed before these antibiotics can be widely used in clinical practice. Further research into these drugs, particularly in combination therapies, may help overcome current limitations and improve pain management strategies.

## Figures and Tables

**Table 1:** This is a table highlighting the current guideline medications to treat neuropathic pain and their mechanism of action, as well as a summary of the antibiotics discussed in the manuscript.

Class/Agent	Mechanism of Action
First-Line Therapies [65,66]	
Anticonvulsants: Gabapentin, Pregabalin.	Bind to the α2δ-1 subunit of voltage-gated calcium channels, which reduces the release of excitatory neurotransmitters in the central nervous system, leading to a decrease in neuronal excitability and pain signaling.
SNRIs: Duloxetine, Venlafaxine.	Inhibit the reuptake of monoamines (serotonin and nor-epinephrine) in the central nervous system, leading to increased levels of these neurotransmitters which then modulate descending pain pathways in the spinal cord, effectively suppressing pain signals and providing analgesic effects.
TCAs: Amitriptyline, Nortriptyline, Desipramine.	Inhibit the reuptake of monoamines at the presynaptic nerve terminals in the spinal cord, leading to increased levels of these neurotransmitters in the synaptic cleft, which can modulate pain signals.
Second-Line Therapies [65,66]	
Capsaicin 8% Patch.	Selectively activating the TRPV1 receptor on sensory nerve fibers, primarily in small-diameter C fibers, leading to a desensitization of these pain-transmitting neurons, effectively blocking pain signals from reaching the central nervous system.
Lidocaine 5% Patch.	Directly blocking voltage-gated sodium channels on damaged nerve fibers and selectively inhibiting the abnormal firing of small unmyelinated C fibers and some A-delta fibers, preventing them from sending pain signals to the brain.
Weak Opioids: Tramadol, Tapentadol.	Act centrally at μ-opioid receptors and act at the spine, inhibiting the reuptake of monoamines.
Third-Line Therapies [65,66]	
Botulinum Toxin A.	Inhibits the release of pain-mediating neurotransmitters, like substance P, CGRP, and glutamate, from sensory nerve terminals, effectively blocking the transmission of pain signals at the peripheral level, reducing pain perception.
Strong Opioids: SR Oxycodone, Morphine.	Binding to the mu-opioid receptor within the central nervous system and the peripheral nervous system, which modulate pain signals.
Experimental Glial Modulator Antibiotics	
Minocycline.	Inhibits NF-κB activation in glial cells, indirectly inhibiting the production of proinflammatory cytokines. Can also modulate autophagy and inhibit the expression of iNOS and cyclooxygenase-2, indirectly inhibiting the production of neurotoxic molecules, like nitric oxide and prostaglandins.
Doxycycline.	Affects the modulation of gene expression in glial cells through GDNF and TH. Inhibits the action of LPS, which acts on glial cells and induces the phosphorylation and activation of the MAPK and NF-κB molecular signaling pathways, indirectly inhibiting the extracellular release of neuroinflammatory cytokines TNF-a and IL-1b. Blunts the production of intracellular ROS, which is accompanied by decreased intracellular glucose and NADPH.
Ceftriaxone.	Stimulates the expression of the glial GLT1, leading to an increase in GLT1 activity, which is associated with delays in neurodegeneration. Modulates oxidative stress and apoptosis signaling by reducing the expression of proapoptotic proteins Bax, Bcl2, caspase 3, and caspase 9.
Azithromycin.	Reduces inflammatory cytokine expression and the M1:M2 microglial phenotype ratio, converting proinflammatory M1 to the anti-inflammatory M2 phenotype. Reduces microglia activation and the expression of several inflammatory biomarkers, including Bax, IL-1b, NF-κB, and superoxide dismutase 1.

Abbreviations: alpha-2-delta (α2δ-1); transient receptor potential vanilloid 1 (TRPV1); calcitonin gene-related peptide (CGRP); kappa-light-chain-enhancer (NF-κB); inducible nitric oxide synthase (iNOS); glial-derived neurotrophic factor (GDNF) and tyrosine hydroxylase (TH); lipopolysaccharide (LPS); mitogen-activated protein kinase (MAPK); tumor necrosis factor-alpha (TNF-a); interleukin-1 beta (IL-1b); reactive oxygen species (ROS); glutamate transporter-1 (GLT1).

## Data Availability

No new data were created or analyzed in this study. Data sharing is not applicable to this article.

## References

[1] Dinakar P., Stillman A.M. (2016). Pathogenesis of Pain. Semin. Pediatr. Neurol..

[2] Fregoso G., Wang A., Tseng K., Wang J. (2019). Transition from Acute to Chronic Pain: Evaluating Risk for Chronic Postsurgical Pain. Pain Physician.

[3] Cheng J. (2018). Mechanisms of Pathologic Pain. Fundamentals of Pain Medicine.

[4] Xu Y., Jiang Y., Wang L., Huang J., Wen J., Lv H., Wu X., Wan C., Yu C., Zhang W. (2019). Thymosin Alpha-1 Inhibits Complete Freund’s Adjuvant-Induced Pain and Production of Microglia-Mediated Pro-inflammatory Cytokines in Spinal Cord. Neurosci. Bull..

[5] Wei Z., Fei Y., Su W., Chen G. (2019). Emerging Role of Schwann Cells in Neuropathic Pain: Receptors, Glial Mediators and Myelination. Front. Cell Neurosci..

[6] Ara I., Maqbool M. (2022). The curious case of Neuropathic Pain and its management: An overview. Open Health.

[7] Tan P.H., Ji J., Yeh C.C., Ji R.R. (2021). Interferons in Pain and Infections: Emerging Roles in Neuro-Immune and Neuro-Glial Interactions. Front. Immunol..

[8] Magni G., Riboldi B., Ceruti S. (2024). Human Glial Cells as Innovative Targets for the Therapy of Central Nervous System Pathologies. Cells.

[9] Paolicelli R.C., Sierra A., Stevens B., Tremblay M.-E., Aguzzi A., Ajami B., Amit I., Audinat E., Bechmann I., Bennett M. (2022). Microglia states and nomenclature: A field at its crossroads. Neuron.

[10] Julius D., Basbaum A.I. (2001). Molecular mechanisms of nociception. Nature.

[11] Scholz J., Woolf C.J. (2007). The neuropathic pain triad: Neurons, immune cells and glia. Nat. Neurosci..

[12] Baral P., Udit S., Chiu I.M. (2019). Pain and immunity: Implications for host defence. Nat. Rev. Immunol..

[13] Breitinger U., Breitinger H.G. (2023). Excitatory and inhibitory neuronal signaling in inflammatory and diabetic neuropathic pain. Mol. Med..

[14] Wang H., Xu C. (2022). A Novel Progress: Glial Cells and Inflammatory Pain. ACS Chem. Neurosci..

[15] Gajtkó A., Bakk E., Hegedűs K., Ducza L., Holló K. (2020). IL-1β Induced Cytokine Expression by Spinal Astrocytes Can Play a Role in the Maintenance of Chronic Inflammatory Pain. Front. Physiol..

[16] Jha M.K., Jeon S., Suk K. (2012). Glia as a Link between Neuroinflammation and Neuropathic Pain. Immune. Netw..

[17] Bannister K., Sachau J., Baron R., Dickenson A.H. (2020). Neuropathic Pain: Mechanism-Based Therapeutics. Annu. Rev. Pharmacol. Toxicol..

[18] Garrido-Mesa N., Zarzuelo A., Gálvez J. (2013). Minocycline: Far beyond an antibiotic. Br. J. Pharmacol..

[19] Cunha B.A., Sibley C.M., Ristuccia A.M. (1982). Doxycycline. Ther. Drug Monit..

[20] Sivamaruthi B.S., Raghani N., Chorawala M., Bhattacharya S., Prajapati B.G., Elossaily G.M., Chaiyasut C. (2023). NF-κB Pathway and Its Inhibitors: A Promising Frontier in the Management of Alzheimer’s Disease. Biomedicines.

[21] Lima I.V., Bastos L.F., Limborço-Filho M., Fiebich B.L., de Oliveira A.C. (2012). Role of prostaglandins in neuroinflammatory and neurodegenerative diseases. Mediat. Inflamm..

[22] Eslami H., Rokhzadi K., Basiri M., Esmaeili-Mahani S., Mahmoodi Z., Haji-Allahverdipoor K. (2024). Direct Interaction of Minocycline to p47phox Contributes to its Attenuation of TNF-α-Mediated Neuronal PC12 Cell Death: Experimental and Simulation Validation. Cell Biochem. Biophys..

[23] Zhou Y.-Q., Liu D.-Q., Chen S.-P., Sun J., Wang X.-M., Tian Y.-K., Wu W., Ye D.-W. (2018). Minocycline as a promising therapeutic strategy for chronic pain. Pharmacol. Res..

[24] Vanelderen P., Rouwette T., Kozicz T., Heylen R., Van Zundert J., Roubos E.W., Vissers K. (2013). Effects of chronic administration of amitriptyline, gabapentin and minocycline on spinal brain-derived neurotrophic factor expression and neuropathic pain behavior in a rat chronic constriction injury model. Reg. Anesth. Pain Med..

[25] Zarei M., Sabetkasaei M., Moini-Zanjani T. (2017). Paradoxical effect of minocycline on established neuropathic pain in rat. EXCLI J..

[26] Chin T.Y., Kiat S.S., Faizul H.G., Wu W., Abdullah J.M. (2017). The Effects of Minocycline on Spinal Root Avulsion Injury in Rat Model. Malays. J. Med. Sci..

[27] Vanelderen P., Van Zundert J., Kozicz T., Puylaert M., De Vooght P., Mestrum R., Heylen R., Roubos E., Vissers K. (2015). Effect of minocycline on lumbar radicular neuropathic pain: A randomized, placebo-controlled, double-blind clinical trial with amitriptyline as a comparator. Anesthesiology.

[28] Martinez V., Szekely B., Lemarié J., Martin F., Gentili M., Ben Ammar S., Lepeintre J.F., de Loubresse C.G., Chauvin M., Bouhassira D. (2013). The efficacy of a glial inhibitor, minocycline, for preventing persistent pain after lumbar discectomy: A randomized, double-blind, controlled study. Pain.

[29] Morrissey E.J., Alshelh Z., Knight P.C., Saha A., Kim M., Torrado-Carvajal A., Zhang Y., Edwards R.R., Pike C., Locascio J.J. (2023). Assessing the potential anti-neuroinflammatory effect of minocycline in chronic low back pain: Protocol for a randomized, double-blind, placebo-controlled trial. Contemp. Clin. Trials.

[30] Guerin C., Laterra J., Masnyk T., Golub L.M., Brem H. (1992). Selective endothelial growth inhibition by tetracyclines that inhibit collagenase. Biochem. Biophys. Res. Commun..

[31] Wang J.-J., Zhang T., Niu D.-B., Wang K., Li K.-R., Xue B., Wang X.-M. (2006). Doxycycline-regulated co-expression of GDNF and TH in PC12 cells. Neurosci. Lett..

[32] Nakamura M., Tsuji O., Bregman B.S., Toyama Y., Okano H. (2011). Mimicking the Neurotrophic Factor Profile of Embryonic Spinal Cord Controls the Differentiation Potential of Spinal Progenitors into Neuronal Cells. PLoS ONE.

[33] Shakhbazau A., Mohanty C., Shcharbin D., Bryszewska M., Caminade A.-M., Majoral J.-P., Alant J., Midha R. (2013). Doxycycline-regulated GDNF expression promotes axonal regeneration and functional recovery in transected peripheral nerve. J. Control. Release.

[34] Yu R., Zheng L., Cui Y., Zhang H., Ye H. (2016). Doxycycline exerted neuroprotective activity by enhancing the activation of neuropeptide GPCR PAC1. Neuropharmacology.

[35] Zhang R., Zhao M., Ji H.J., Yuan Y.H., Chen N.H. (2013). Study on the dynamic changes in synaptic vesicle-associated protein and axonal transport protein combined with LPS neuroinflammation model. ISRN Neurol..

[36] Cunningham C. (2013). Microglia and neurodegeneration: The role of systemic inflammation. Glia.

[37] Santa-Cecília F.V., Socias B., Ouidja M.O., Sepulveda-Diaz J.E., Acuña L., Silva R.L., Michel P.P., Del-Bel E., Cunha T.M., Raisman-Vozari R. (2016). Doxycycline Suppresses Microglial Activation by Inhibiting the p38 MAPK and NF-kB Signaling Pathways. Neurotox. Res..

[38] Junior N.C.F., Pereira M.d.S., Francis N., Ramirez P., Martorell P., González-Lizarraga F., Figadère B., Chehin R., Del Bel E., Raisman-Vozari R. (2021). The Chemically-Modified Tetracycline COL-3 and Its Parent Compound Doxycycline Prevent Microglial Inflammatory Responses by Reducing Glucose-Mediated Oxidative Stress. Cells.

[39] Fodelianaki G., Lansing F., Bhattarai P., Troullinaki M., Zeballos M.A., Charalampopoulos I., Gravanis A., Mirtschink P., Chavakis T., Alexaki V.I. (2019). Nerve Growth Factor modulates LPS—Induced microglial glycolysis and inflammatory responses. Exp. Cell Res..

[40] Nascimento G.C., Vivanco-Estela A.N., Ferrié L., Figadere B., Raisman-Vozari R., Michel P.P., Del Bel E. (2024). Anti-nociceptive effects of non-antibiotic derivatives of demeclocycline and doxycycline against formalin-induced pain stimulation. Eur. J. Pharmacol..

[41] Auer G.K., Weibel D.B. (2017). Bacterial Cell Mechanics. Biochemistry.

[42] Rothstein J.D., Patel S., Regan M.R., Haenggeli C., Huang Y.H., Bergles D.E., Jin L., Hoberg M.D., Vidensky S., Chung D.S. (2005). Beta-lactam antibiotics offer neuroprotection by increasing glutamate transporter expression. Nature.

[43] Hu Y., Li W., Lu L., Cai J., Xian X., Zhang M., Li Q. (2010). An anti-nociceptive role for ceftriaxone in chronic neuropathic pain in rats. Pain.

[44] Chen Z., He Y., Wang Z.J. (2012). The beta-lactam antibiotic, ceftriaxone, inhibits the development of opioid-induced hyperalgesia in mice. Neurosci Lett..

[45] Gunduz O., Oltulu C., Buldum D., Guven R., Ulugol A. (2011). Anti-allodynic and anti-hyperalgesic effects of ceftriaxone in streptozocin-induced diabetic rats. Neurosci. Lett..

[46] Inquimbert P., Bartels K., Babaniyi O.B., Barrett L.B., Tegeder I., Scholz J. (2012). Peripheral nerve injury produces a sustained shift in the balance between glutamate release and uptake in the dorsal horn of the spinal cord. Pain.

[47] Luo X., He T., Wang Y., Wang J.-L., Yan X.-B., Zhou H.-C., Wang R.-R., Du R., Wang X.-L., Chen J. (2020). Ceftriaxone Relieves Trigeminal Neuropathic Pain Through Suppression of Spatiotemporal Synaptic Plasticity via Restoration of Glutamate Transporter 1 in the Medullary Dorsal Horn. Front. Cell Neurosci..

[48] Amin B., Hajhashemi V., Abnous K., Hosseinzadeh H. (2014). Ceftriaxone, a beta-lactam antibiotic, modulates apoptosis pathways and oxidative stress in a rat model of neuropathic pain. BioMed Res. Int..

[49] Amin B., Hajhashemi V., Hosseinzadeh H. (2015). Minocycline potentiates the anti-hyperalgesic effect of ceftriaxone in CCI-induced neuropathic pain in rats. Res. Pharm. Sci..

[50] Pottabathini R., Kumar A., Bhatnagar A., Garg S., Ekavali E. (2016). Ameliorative potential of pioglitazone and ceftriaxone alone and in combination in rat model of neuropathic pain: Targeting PPARγ and GLT-1 pathways. Pharmacol. Rep..

[51] Macaluso A., Bernabucci M., Trabucco A., Ciolli L., Troisi F., Baldini R., Gradini R., Battaglia G., Nicoletti F., Collini S. (2013). Analgesic effect of a single preoperative dose of the antibiotic ceftriaxone in humans. J. Pain.

[52] Ware M.A., Bennett G.J. (2014). Case report: Long-standing complex regional pain syndrome relieved by a cephalosporin antibiotic. Pain.

[53] Shinkai M., Henke M.O., Rubin B.K. (2008). Macrolide antibiotics as immunomodulatory medications: Proposed mechanisms of action. Pharmacol. Ther..

[54] Amantea D., Certo M., Petrelli F., Bagetta G. (2016). Neuroprotective Properties of a Macrolide Antibiotic in a Mouse Model of Middle Cerebral Artery Occlusion: Characterization of the Immunomodulatory Effects and Validation of the Efficacy of Intravenous Administration. Assay Drug Dev. Technol..

[55] Gensel J.C., Kopper T.J., Zhang B., Orr M.B., Bailey W.M. (2017). Predictive screening of M1 and M2 macrophages reveals the immunomodulatory effectiveness of post spinal cord injury azithromycin treatment. Sci. Rep..

[56] Kopper T.J., McFarlane K.E., Bailey W.M., Orr M.B., Zhang B., Gensel J.C. (2019). Delayed Azithromycin Treatment Improves Recovery After Mouse Spinal Cord Injury. Front. Cell Neurosci..

[57] Gensel J.C., Donahue R.R., Bailey W.M., Taylor B.K. (2019). Sexual Dimorphism of Pain Control: Analgesic Effects of Pioglitazone and Azithromycin in Chronic Spinal Cord Injury. J. Neurotrauma..

[58] Rismanbaf A., Afshari K., Ghasemi M., Badripour A., Haj-Mirzaian A., Dehpour A.R., Shafaroodi H. (2022). Therapeutic Effects of Azithromycin on Spinal Cord Injury in Male Wistar Rats: A Role for Inflammatory Pathways. J. Neurol. Surg. A Cent. Eur. Neurosurg..

[59] Zloto O., Zahavi A., Richard S., Friedman-Gohas M., Weiss S., Goldenberg-Cohen N. (2022). Neuroprotective Effect of Azithromycin Following Induction of Optic Nerve Crush in Wild Type and Immunodeficient Mice. Int. J. Mol. Sci..

[60] Mahaling B., Pandala N., Wang H.C., Lavik E.B. (2022). Azithromycin Protects Retinal Glia Against Oxidative Stress-Induced Morphological Changes, Inflammation, and Cell Death. ACS Bio Med Chem Au.

[61] Kim T.H., Kim H.I., Kim J., Park M., Song J.H. (2010). Effects of minocycline on Na+ currents in rat dorsal root ganglion neurons. Brain Res..

[62] do Amaral L., dos Santos N.A.G., Sisti F.M., Del Bel E., dos Santos A.C. (2021). The antibiotic doxycycline mimics the NGF signaling in PC12 cells: A relevant mechanism for neuroprotection. Chem. Biol. Interactions.

[63] Tikhonova M.A., Amstislavskaya T.G., Ho Y.J., Akopyan A.A., Tenditnik M.V., Ovsyukova M.V., Bashirzade A.A., Dubrovina N.I., Aftanas L.I. (2021). Neuroprotective Effects of Ceftriaxone Involve the Reduction of Aβ Burden and Neuroinflammatory Response in a Mouse Model of Alzheimer’s Disease. Front. Neurosci..

[64] Venditto V.J., Haydar D., Abdel-Latif A., Gensel J.C., Anstead M.I., Pitts M.G., Creameans J., Kopper T.J., Peng C., Feola D.J. (2021). Immunomodulatory Effects of Azithromycin Revisited: Potential Applications to COVID-19. Front. Immunol..

[65] Bates D., Schultheis B.C., Hanes M.C., Jolly S.M., Chakravarthy K.V., Deer T.R., Levy R.M., Hunter C.W. (2019). A Comprehensive Algorithm for Management of Neuropathic Pain. Pain Med..

[66] Fornasari D. (2017). Pharmacotherapy for Neuropathic Pain: A Review. Pain Ther..

